# Whole-genome bisulfite sequencing reveals the function of DNA methylation in the allotransplantation immunity of pearl oysters

**DOI:** 10.3389/fimmu.2023.1247544

**Published:** 2023-10-03

**Authors:** Zefeng Gu, Jingmiao Yang, Jinzhao Lu, Min Yang, Yuewen Deng, Yu Jiao

**Affiliations:** ^1^Fishery College, Guangdong Ocean University, Zhanjiang, China; ^2^Pearl Breeding and Processing Engineering Technology Research Centre of Guangdong Province, Zhanjiang, China; ^3^Guangdong Science and Innovation Center for Pearl Culture, Zhanjiang, China; ^4^Guangdong Provincial Key Laboratory of Aquatic Animal Disease Control and Healthy culture, Zhanjiang, China

**Keywords:** allograft transplantation, DNA methylation, WGBS, nAChR, Pinctada fucata martensii

## Abstract

**Introduction:**

In the pearl culture industry, a major challenge is the overactive immunological response in pearl oysters resulting from allotransplantation, leading to shell-bead rejection and death. To better understand the molecular mechanisms of postoperative recovery and the regulatory role of DNA methylation in gene expression, we analyzed the changes in DNA methylation levels after allotransplantation in pearl oyster *Pinctada fucata martensii*, and elucidated the regulatory function of DNA methylation in promoter activity of *nicotinic acetylcholine receptor* (*nAChR*) gene.

**Methods:**

We constructed nine DNA methylomes at different time points after allotransplantation and used bisulfite genomic sequencing PCR technology (BSP) to verify the methylation status in the promoter of *nAChR*. We performed Dual luciferase assays to determine the effect of the dense methylation region in the promoter on transcriptional activity and used DNA pull-down and mass spectrometry analysis to assess the capability of transcription factor binding with the dense methylation region.

**Result:**

The DNA methylomes reveal that CG-type methylation is predominant, with a trend opposite to non-CG-type methylation. Promoters, particularly CpG island-rich regions, were less frequently methylated than gene function elements. We identified 5,679 to 7,945 differentially methylated genes (DMGs) in the gene body, and 2,146 to 3,385 DMGs in the promoter at each time point compared to the pre-grafting group. Gene ontology and pathway enrichment analyses showed that these DMGs were mainly associated with “cellular process”, “Membrane”, “Epstein-Barr virus infection”, “Notch signaling pathway”, “Fanconi anemia pathway”, and “Nucleotide excision repair”. Our study also found that the DNA methylation patterns of the promoter region of *nAChR* gene were consistent with the DNA methylomics data. We further demonstrated that the dense methylation region in the promoter of *nAChR* affects transcriptional activity, and that the methylation status in the promoter modulates the binding of different transcription factors, particularly transcriptional repressors.

**Conclusion:**

These findings enhance our understanding of the immune response and regulation mechanism induced by DNA methylation in pearl oysters after allotransplantation.

## 1 Introduction

Epigenetic mechanisms, which include chromosome remodeling (1), histone modification (2), DNA methylation (3), and noncoding RNA regulation (4), play a critical role in the immune system by regulating gene expression (5). Among these mechanisms, DNA methylation is a dynamic epigenetic modification that is vital in the regulation of immune responses and transcriptional control (6). This process entails the addition of a methyl group to the C5 position of a specific cytosine (C) and is catalyzed by DNA methyltransferase enzymes that primarily target the cytosine of the CG dinucleotide in the vertebrate genome (7). In mammals, DNA methylation in the promoter region is a critical mechanism that impedes transcription initiation (8). Genes are regulated by changing the methylation status of multiple CG sites in the promoter region, thus decreasing or improving the transcription efficiency of the promoter to affect the level of gene expression. In many lower organisms, including metazoans such as *Caenorhabditis elegans* and *Drosophila melanogaster*, DNA methylation is absent and not always essential for gene regulation (9). In addition, mollusk genome exhibits “mosaic” methylation patterns that selectively target specific genomic elements (10).

DNA methylation is a crucial epigenetic modification in organisms and a dynamic process of methylation and demethylation. This modification has been linked to changes in gene expression through the recruitment of methylated DNA-binding proteins (11, 12). Methylation of CG sites in promoter regions serves as an epigenetic marker that leads to gene expression silencing, even in the presence of transcriptional factors. The methylation of gene bodies remains inconclusive, with the highest levels of methylation potentially contributing to increased gene expression (13). Recent studies have shed light on the critical role of DNA methylation in regulating innate immune responses. For instance, Kim et al. (14) demonstrated that the hypomethylation of the promoter of toll-like receptor 4 (TLR4) activates nuclear factor kappa-B (NF-κB) expression, thereby modulating immune responses. Similarly, Pacis et al. (15) showed that within 24 h of *Mycobacterium tuberculosis* infection, the distal enhancers of dendritic cells rapidly undergo demethylation and activate several immune transcription factors, including NF-κB and members of the interferon regulatory factor family. These findings provide compelling evidence for the role of DNA methylation in regulating innate immune responses. In *European sea bass*, a slight ocean temperature difference can change DNA methylation and thus induce the expression of genes involved in stress and heat shock response (16). In colonial ascidian *Didemnum vexillum*, temperature stress remarkably alters DNA methylation patterns (17). Gavery et al. (18) observed that DNA methylation could regulate the functional genes of Pacific oyster *Crassostrea gigas* in response to environmental stresses. All these studies highlighted the dynamic nature of DNA methylation in response to environmental stimuli and underscore its critical role in regulating gene expression and survival.

**Figure 1:**
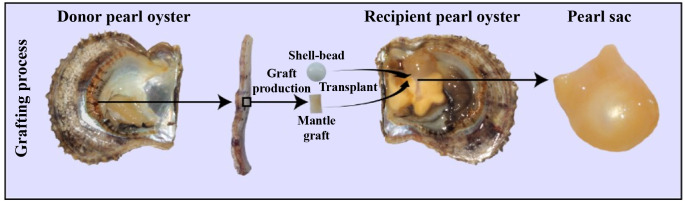
Artificial grafting process. During pearl production, a mantle graft (approximately 4 mm2) is extracted from a donor oyster and then transplanted along with a spherical shell-bead (approximately 6 mm in diameter) into the gonad of a host oyster. Subsequently, the host oyster develops a pearl sac that envelops the pearl-nuclei, leading to the formation of a pearl.

*Pinctada fucata martensii*, a species of pearl oyster, is of tremendous economic significance in saltwater pearl production (19). For the simulation of natural pearl formation, modern pearl production involves the surgical transplantation of a mantle graft (measuring approximately 4 mm^2^) from a donor oyster and a spherical shell-bead (about 6 mm in diameter) into the gonad of a host oyster (20). Generally, the transplanted mantle graft is from different individuals of the same species, which is called allotransplantation; xenotransplantation means the transplanted mantle graft are from different species (21). After the surgical transplantation, the introduction of foreign bodies and pathogens by the transplantation surgery elicits a robust immune response in the recipient oyster, which can result in shell-bead rejection, failed pearl sac formation, and even mortality (22). The recipient oyster forms a pearl sac that encases the shell-bead, ultimately forming a pearl through the continuous secretion of nacre (**Figure 1**) (23). Transcriptomes analysis revealed many genes involved in the immune regulation of pearl oysters. Our previous research found that nAChR could regulate the inflammatory response induced by transplantation by modulating the apoptosis and proliferation of hemocytes and repairing damaged DNA in pearl oysters (24). nAChRs belong to a superfamily of pentameric ligand-gated ion channel proteins (25). In mammals, nAChRs are widely expressed in the nervous system where they regulate neurotransmitter release, cell excitability, and neuronal integration. These functions are crucial for maintaining physiological homeostasis related to fatigue, pain processing, immune response, and stress (26). In the organs of bivalve mollusks, such as *Chlamys farreri*, two *nAChR* genes have been detected and their expression increases after stimulation with lipopolysaccharides and tumor necrosis factor-alpha, indicating their role in immunomodulation (27). In oysters and scallops, ACh and nAChRs regulate immune response possibly through the neuroendocrine-immune system (28, 29).

In this study, we performed the whole-genome bisulfite sequencing (WGBS) of hemocytes before and after allotransplantation to investigate the role of methylation in the allotransplantation immunity of pearl oyster *P. f. martensii*. We also verified the methylation status in the promoter regions of *nAChR* genes and analyzed the effect of methylation on promoter activity and transcription factor binding to elucidate the promoter-regulating mechanism of DNA methylation in pearl oysters. The results of this study will expand our understanding of the methylome of pearl oysters and provide insight into the immunomodulatory mechanisms of methylation in mollusks.

## 2 Materials and methods

### 2.1 Sample preparation

Pearl oyster *P. f. martensii* was sourced from Dajing Village, Xuwen County, Zhanjiang, Guangdong Province, China. The specimens were 1.5 years old and had a mean shell length of 60.35 ± 6.11 mm at the commencement of the experiment (30). Prior to the transplant procedure, each group of 50 pearl oysters was placed in a 30 cm-diameter cage and subjected to pregrafting conditioning, whereby the lines were raised to the water’s surface during the hottest part of the day for 8 days. Following surgical implantation, hemolymph was collected from the adductor muscles of at least 10 host pearl oysters from each group at various time points (6 and 12 h and 1, 3, 6, 12, 18, and 30 days) using 1 mL syringes. The collected fluid was subsequently centrifuged at 3500 rpm for 5 min to separate the hemocytes from the sediment at the bottom of the tube. Finally, the hemocytes were immersed in 75% ethanol and stored at 4°C in a refrigerator. The control group (Con) comprised pearl oysters that underwent pregrafting conditioning but not actual transplantation.

### 2.2 DNA extraction and library construction

Individual DNA samples were extracted using the TIANamp genomic DNA kit (TIAN GEN, China) and eight individuals were mixed in equal proportions. For the construction of the typical WGBS library, the DNA was fragmented *via* sonication using a Bioruptor (Diagenode, Belgium) to an average size of approximately 250 bp. The fragmented DNA was then subjected to blunt-ending, addition to the 3'-end, and adaptor ligation using methylated adaptors to protect it from bisulfite conversion in accordance with the manufacturer’s instructions. The ligated DNA was then bisulfite converted using the EZ DNA methylation gold kit (ZYMO). Different insert-size fragments were excised from the same lane of a 2% TAE agarose gel, purified using the QIAquick gel extraction kit (Qiagen), and amplified by PCR. Finally, sequencing was performed using HighSeq 4000 or other Illumina platforms.

### 2.3 DNA-seq data analysis

Adaptor sequences, contaminants, and low-quality reads were initially removed from the raw data following the delivery of the sequencing data. The reference genome was then mapped using the clean data with BSMAP (31), and a sufficient amount of clean data was ensured. The reference genome utilized in this study was based on prior research (32). The alignment underwent a quality check. The methylation information for cytosine throughout the whole genome was subsequently obtained using the uniquely mapped data. The cytosine data were then employed for generic and customized bioinformatics analyses.

### 2.4 Methylation level

The methylation level of the genome provides insight into the overall properties of the methylome and can be calculated by determining the proportion of reads supporting methylation to the reads covering specific cytosine sites. The total number of reads covering the cytosine was divided by the sum of reads covering each methylated cytosine (mC), which was equal to the mC/C ratio for each reference cytosine (33). The formula was as follows:


$$Rm_{\text{average}}=\frac{Nm_{\text{all}}}{Nm_{\text{all}}+Nnm_{\text{all}}}*100\%$$

where Nm represents the reads number of mC, Nnm represents the reads number of nonmethylation reads, and Rm represents the methylation level of methylated cytosine.

### 2.5 Differentially methylated region detection

Windows with at least five CG (CHG or CHH) sites, a twofold change in methylation level, and Fisher test *P< 0.05* were selected to identify putative differentially methylated regions (DMRs) between the control and other groups. Neither group should be hypomethylated during DMR discovery. If the genomic area from the beginning of an upstream DMR to the end of a downstream DMR displayed twofold methylation level variations between Con and other groups with a *P< 0.05*, then the two neighboring DMRs were deemed interdependent and combined into one continuous DMR. The two DMRs were considered independent in all other respects. The final dataset of DMRs comprised those of DMRs independent of one another after repeatedly combining the interdependent DMRs.

### 2.6 Degree of difference in methylation level

For the comparison of DMR methylation levels among the samples, CIRCOS was used to determine differences in a methylated cytosine (mCG, mCHG, or mCHH) between the two groups. The formula was as follows:


$$\text{degree of difference}=\frac{{\text{log}}_{2}\text{Rm}1}{{\text{log}}_{2}\text{Rm}2'}$$

where Rm1 and Rm2 indicate the methylation level of methylated cytosine for Con and other groups, respectively. When Rm1 (or Rm2) is 0, the value is replaced with 0.001.

### 2.7 Function enrichment of DMR-related genes

DMR-related genes from both groups were evaluated using the Kyoto Encyclopedia of Genes and Genomes (KEGG) and Gene Ontology (GO) databases. The DMGs were mapped to the GO term database (http://www.geneontology.org/) to determine the number of genes associated with each GO term (34), and a hypergeometric test was used to calculate the GO term with the highest enrichment (*P*< 0.05). KEGG (35) is a public database on pathways (http://www.genome.jp/kegg), and hypergeometric tests were conducted to analyze the significant enrichment of DMGs in KEGG pathways. Pathways with a *P< 0.05* were considered highly enriched in DMGs.

### 2.8 Online website prediction

Berkeley Drosophila Genome Project was used to predict the promoter activity and transcription factor binding sites of promoter sequences, and Match 1.0 Public was utilized to identify transcription factor binding sites in DNA sequences.

Berkeley Drosophila Genome Project: Neural Network Promoter Prediction (https://www.fruitfly.org/seq tools/promoter.html) is a website that can predict the promoter activity of the promoter sequence. The training and test sets of human and *Drosophila melanogaster* promoter sequences are accessible to the community for testing transcription start site predictors (36). These sites also contain our representative, standardized data sets of human and *Drosophila melanogaster* genes. Match 1.0 Public (http://gene-regulation.com/cgi-bin/pub/programs/match/bin/match.cgi) is a weight matrix-based software that utilizes the TRANSFAC® Public 6.0 library of positional weight matrices to identify transcription factor binding sites in DNA sequences and predict the transcription factor binding site of promoter sequences (37).

### 2.9 Bisulfite sequencing polymerase chain reaction

DNA treatment with sodium bisulfite was performed using the EZ DNA Methylation Kit (Zymo Research, USA) in accordance with the manufacturer’s protocol. Following modification, the DNA samples were diluted in 10 mL of distilled water and immediately used for bisulfite sequencing PCR (BSP). BSP primers were designed with Methyl Primer Express v1.0, and the sequences of the PCR primers utilized for amplifying the targeted products are presented in **Table 1**. Hot start DNA polymerase (Zymo Taq TM Premix, Zymo Research, USA) was utilized for BSP performed in 50 mL of reaction volume containing 200 ng/50 mL genomic DNA, 0.3–1 mM each primer, and 25 mL of Zymo Taq TM Premix. PCR amplification was carried out using a DNA Engine Thermal Cycler (Bio-Rad, USA) with the following program: initial denaturation at 95°C for 10 min, followed by 40 cycles of denaturation for 30 s at 95°C, annealing for 30 s at 57.5°C, and extension for 30 s at 72°C, with a final extension at 72°C for 10 min. The PCR products were subsequently gel purified using the Gel Purification Kit (Sangon, Shanghai, China), and the purified fragments were subcloned into the pMD-19T Vector (TaKaRa, China). Ten positive clones for each subject were randomly selected for sequencing (Sangon, Shanghai, China), and the final sequence results were processed using the BIQ-Analyzer.

**Table 1:** Primers used in BSP and promoter clone.

Primer name	Primer sequences	Purpose
*nAChR* - F	TTTGTATTTATTTGGAAAGTTTATGTGTT	BSP
*nAChR* - R	CCCTCATTTTTCCACTACTAATCTCTT	BSP
*nAChR* - F1	TCATTCGGAAAGTCCATGTGT	clone
*nAChR* - F2	TATCGGGCCCTTTTTTACGT	clone
*nAChR* - R	ACAATCTCCCATCCCTCCAT	clone

### 2.10 Construction of promoter vector of *nAChR*

DNA samples were extracted from pearl oysters using the TIANamp genomic DNA kit (TIAN GEN, China). Six primers were designed (**Table 1**) and genomic DNA was used as a template for PCR to obtain fragments with and without the dense methylation regions of the *nAChR* promoters. The constructs included −1080 bp to −1 bp and −921 bp to −1 bp (relative to the transcription start site) from the *nAChR* promoter. PrimerSTAR HS DNA polymerase (2.5 U/mL) (Takara, Dalian, China) was used to amplify the DNA fragments, which were then digested with Xho I and Hind III restriction enzymes. The PCR products were then inserted into the pGL3-base vector (Promega, Madison, WI, USA) containing the luciferase reporter gene in accordance with the manufacturer’s instructions.

### 2.11 Plasmid transfection and dual luciferase reporter assay

When the HEK-293 T cells reached 90% confluence, the proliferation medium was removed and the HEK-293 T cells were rinsed with phosphate-buffered saline and treated with 0.5% trypsin for 1 min to detach them from the plate. The HEK-293 T cells were then collected, centrifuged, diluted in a proliferation medium prepared with DMEM without penicillin/streptomycin (pen/strep), and split onto one 48-well plate at a density of approximately 10^4^ cells per well.

The following day, the cells were transfected with Lipofectamine 3000 (Invitrogen, Carlsbad, CA, USA) as per the manufacturer’s instructions. For each well, a mixture of 0.5 μL of Lipofectamine 3000, 0.5 μL of P3000, and 300 ng of DNA was introduced in 100 μL of FBS-free and pen/strep-free Opti-MEMI medium (Promega, Madison, WI, USA) and incubated for 15 min. The pRL-TK plasmid vector (Promega, Madison, WI, USA) was cotransfected with the reporter construct to normalize the transfection efficiency.

The experiments were performed in triplicate for each construct. The relative activities of the fragments with or without dense methylation region in the promoter were analyzed using the Dual Luciferase Reporter Assay System (Promega, Madison, WI, USA) as per the manufacturer’s protocol. The cells were harvested 24 h post-transfection. Firefly and Renilla luciferase activities were measured using the Dual-Luciferase Reporter Assay System (Promega, Madison, WI, USA) and EnSipre Multifunctional Enzyme Labeler (PerkinElmer, Waltham, MA, USA), respectively.

The firefly luciferase activities were normalized by the Renilla luciferase activities in each well, and the data were presented as the average of three replicates in the results section.

### 2.12 DNA pull-down assay and mass spectrometry

DNA pull-down assays were conducted using biotinylated DNA probes synthesized with Desthio-Biotin-TEG at the 5' end and obtained from Sangon (Shanghai, China). Methylation probes were prepared by methylating the DNA using M.SssI (New England Biolabs). For the preparation of the methylation and unmethylation probes, two biotin-labeled probes were synthesized and combined to form a double-stranded DNA. Nucleoproteins were extracted from pearl oyster hemolymph following the instructions provided in the Nucleoprotein Extraction Kit (Sangon, Shanghai, China).

For the pull-down assays followed by mass spectrometry, 5 μL of each nuclear protein sample was incubated with the DNA probe for 60 min at 4°C with rotation. Afterward, 120 μL of SA magnetic beads were added to the mixture, which was centrifuged at 12,000 rpm for 1 min at 4°C. The supernatant was discarded, and the bound proteins were washed twice in 800 μL of IP lysis solution. For mass spectrometry, the proteins captured during DNA pull-downs were stored in a refrigerator at −80°C. Peptides were then extracted, desalted using StageTips, and analyzed using an Orbitrap Velos mass spectrometer.

## 3 Results

### 3.1 DNA methylation mapping and patterns

After allotransplantations at different times (6 and 12 h and 1, 3, 6, 12, 18, and 30 days), the DNA methylation profiles of hemocytes were successfully constructed for eight allotransplantation groups and one control group. We generated an average of 41.31 G raw bases for the nine groups. After data filtering, the mapped reads could be used for subsequent analysis with rates ranging from 61.66% to 64.92%. We observed DNA methylation in three sequence contexts, CG, CHG, and CHH, with an overall proportion of 100%. The distribution of the three types of methylation was consistent across all groups, with an approximate overall distribution of 90.08%–93.11% for mCG, 1.58%–2.08% for mCHG, and 5.31%–7.84% for mCHH (**Supplementary Figure S1**).

### 3.2 Characteristics analysis of methylation levels

**Figure 2:**
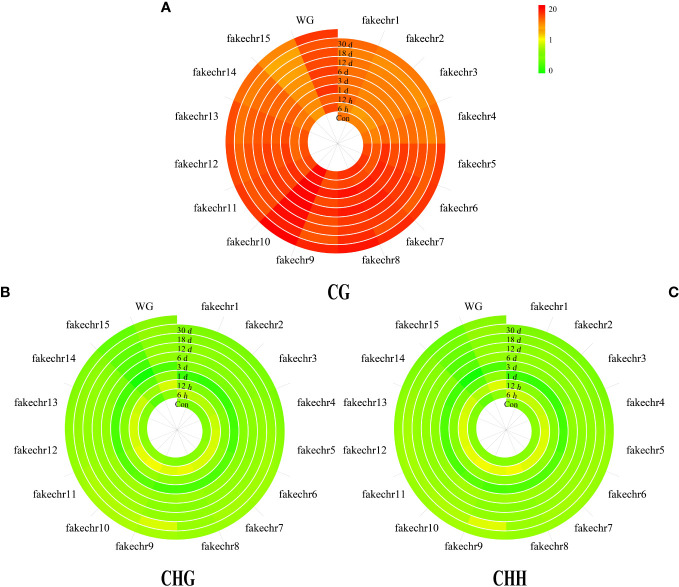
The plot of genome chromosome methylcytosine map. **(A)** CG type. **(B)** CHG type. **(C)** CHH type. The methylation levels of each window are described using colors (the window from green to red represents the methylation level from 0 to 20).

To further investigate the methylation pattern, we plotted chromosome methylation maps for each sample. Our results showed that the methylation level of CG was higher than that of CHG and CHH in all chromosomes, with the highest methylation level observed in fakechr 10 and the lowest in fakechr 15 (**Figure 2**, **Supplementary Material 1**). CHG and CHH types showed the highest methylation levels at 6 h, and CG types had the lowest methylation levels.

### 3.3 Methylation levels in different gene regions

**Figure 3:**
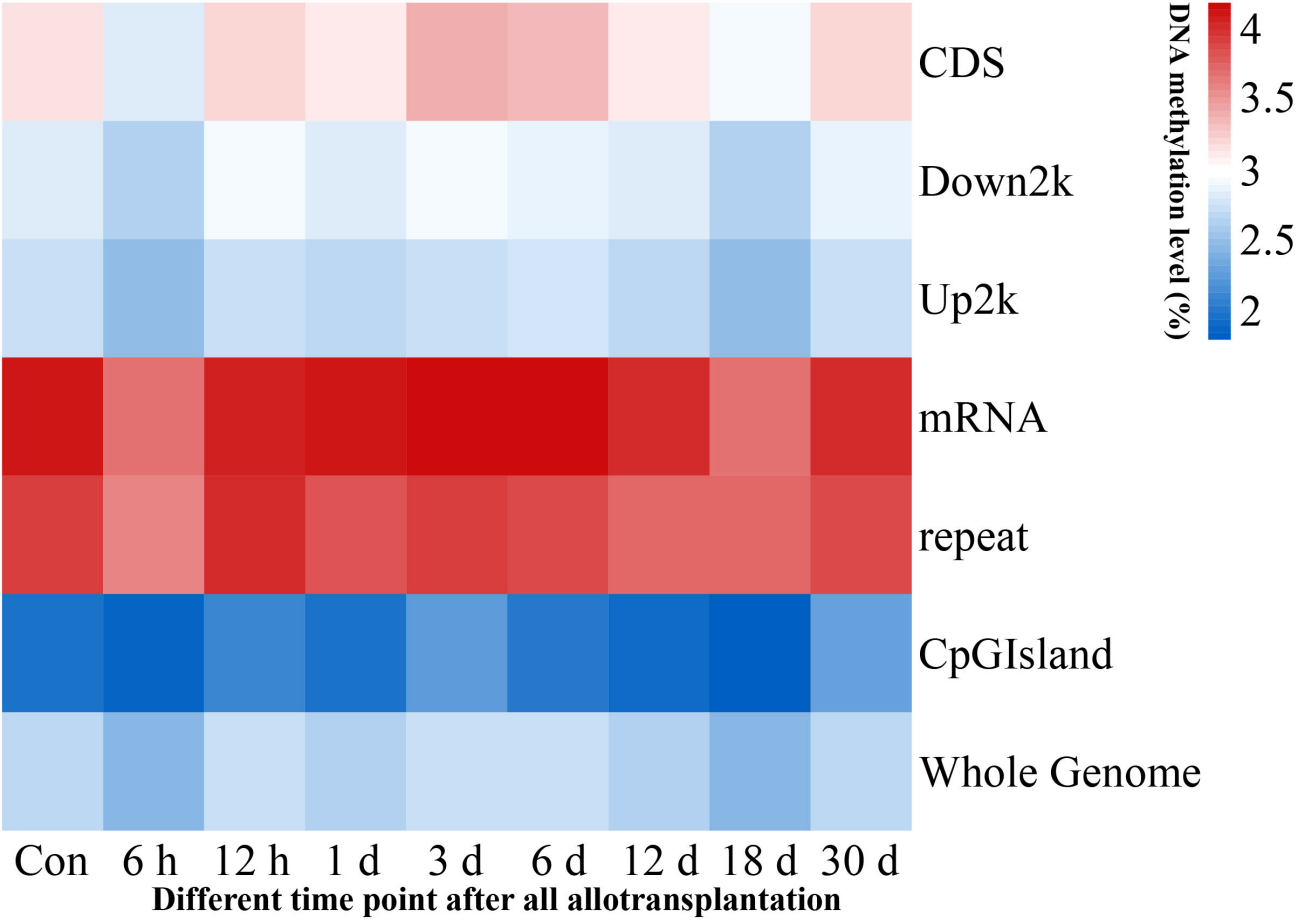
Methylation levels in genome-wide transcriptional and regulatory elements. Each window is represented with colors, where the color intensity corresponds to the methylation level of the respective gene region at a given time point, normalized by the total sum of methylation levels across all time points.

We investigated the methylation levels in different gene regions and observed remarkable differences between transcriptional and regulatory regions. Methylated cytosines were primarily found in the mRNA and repeat sequence regions, and the upstream 2000 bp area and CpG island region had the lowest levels of methylation. Our findings also showed that pearl oyster hemolymph demethylated at 6 h and 18 days after allotransplantation, followed by the restoration of methylation at 12 h and 30 days in all transcriptional and regulatory elements (**Figure 3**, **Supplementary Material 2**).

To further explore the changing trends of methylation levels in different transcriptional elements, we divided mC into specific gene features, namely, the transcription start site (TSS) upstream of the 2000 bp sequence (upstream), first exon, first intron, exon within the protein-coding region (internal exon), intron within the protein-coding region (internal intron), last exon, and downstream 2000 bp sequence (downstream). Our results were consistent with previous research (38), showing opposite trends between non-CG types and CG types. We also found that the methylation levels of CG type in the internal exon increased from 6 h to 12 h, and those of the non-CG type decreased. Difference in methylation for 6 h and 18 days compared with the other times was mainly observed in the upstream and protein-coding regions (**Supplementary Figure S2**).

### 3.4 Analysis of methylation sequence preferences

To investigate the relationship between sequence context and methylation preference in pearl oysters, we assessed the methylation percentage of all possible 7-mer sequences where the methylated cytosine is located in the fourth position. This analysis allowed for the examination of three nucleotides upstream of CG, CHG, and CHH methylation sites. Our results indicated that CG and non-CG type methylation sites had a preference for thymine (T) in the upstream sequences. Adenine (A) was most commonly observed in the downstream bases immediately adjacent to the methylated cytosine site of the non-CG type. Furthermore, we observed that the methylation preference differences between different time points after allotransplantation were the most pronounced at the second site upstream or downstream of non-CG methylation, corresponding to the change between A and T (**Supplementary Figure S3**).

### 3.5 Statistical analysis of differentially methylated regions

**Figure 4:**
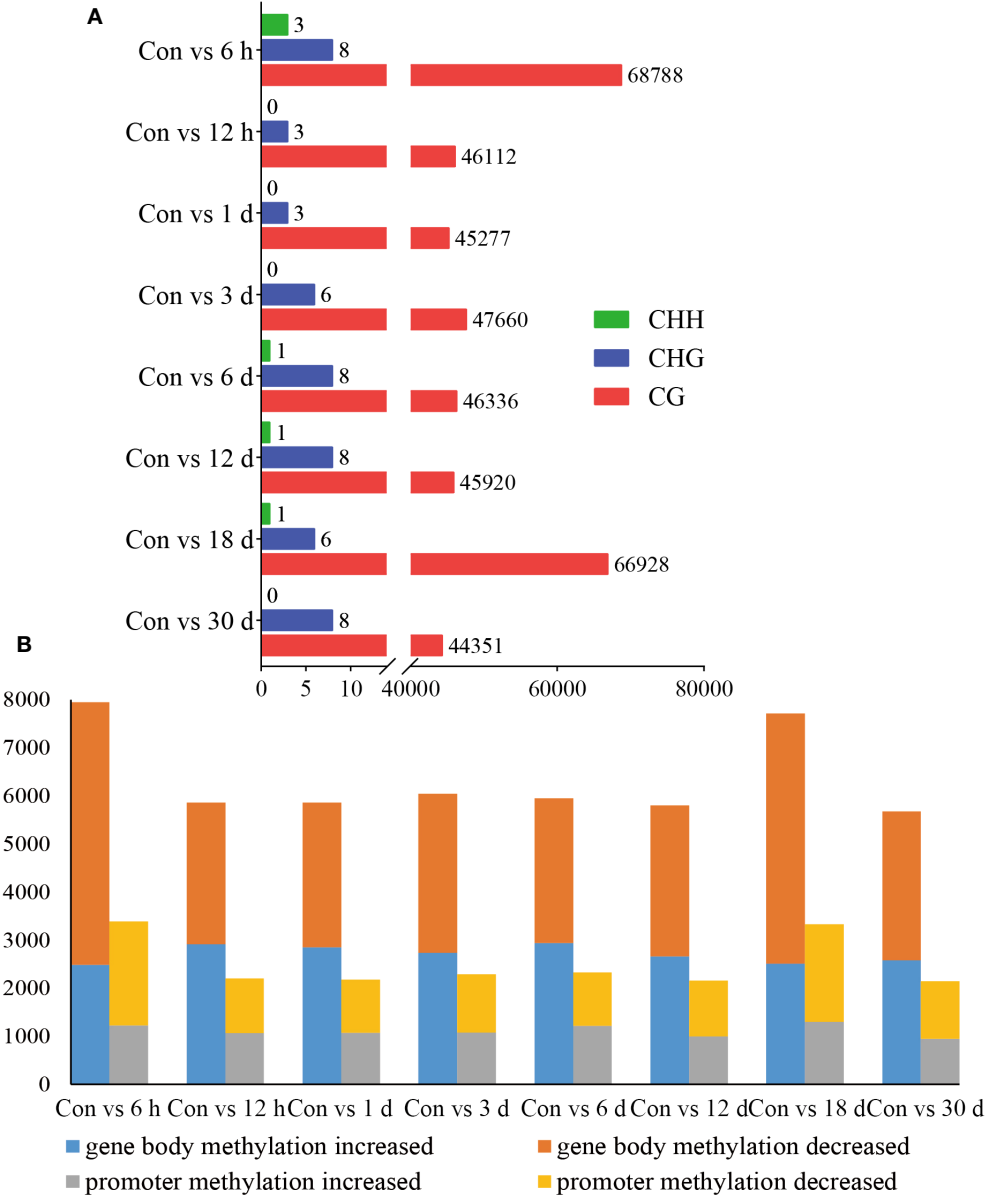
Statistical results of methylation differences. **(A)** DMRs between Con and different times after grafting. The horizontal axis is the number of DMRs and the vertical axis is the type of different groups. **(B)** DMGs between Con and different times after grafting. The horizontal axis is the type of comparison in different groups, each of which is analyzed for gene body and promoters, and the vertical axis is the number of enriched DMGs.

DMRs are DNA segments with distinct DNA methylation patterns. To identify differential methylation, we comprehensively explored the changes in the methylation status of hemocytes in pearl oysters after allotransplantation. We quantified the number of DMRs between different groups and found that the non-CG type exhibited a relatively lower number of DMRs than the CG type, suggesting that the CG type is the primary form of methylation. Our data revealed that the highest number of DMRs between the control group and those at 6 h and 18 days post-allotransplantation (**Figure 4A**, **Supplementary Material 3**).

We then identified the genes where DMRs reside, referred to as DMR-associated genes (DMGs). Our analysis showed that the number of DMGs found in the gene body (5679–7945) was higher than that in the promoter (2146–3385). The maximum number of DMGs was detected at 6 h after allotransplantation, which is consistent with the pattern observed for DMRs (**Figure 4B**, **Supplementary Material 4**).

We selected the Con and 6 h groups for incorporation into the statistical analysis of methylation changes within the gene regions for generating a boxplot in **Supplementary Figure S4**. This plot roughly illustrates similar methylation distinctions between promoter and gene body regions, while also highlighting greater measures of dispersion in DNA methylation differences for DMGs in promoter regions.

### 3.6 Functional analysis of DMGs

**Figure 5:**
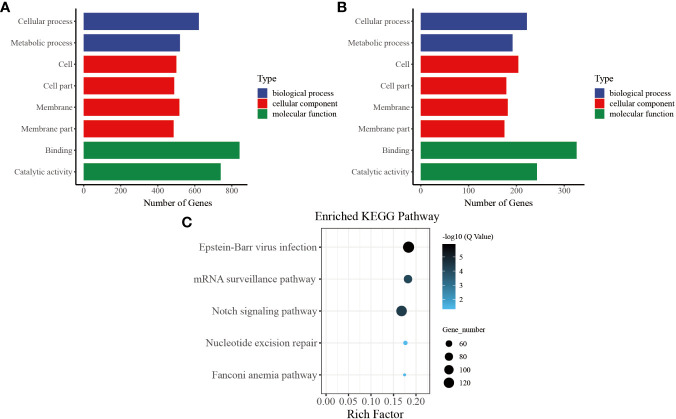
Common entries for functional analysis of CG-type DMGs at different times. **(A)** Gene ontology in the gene body, the x-axis represents three GO domains, while the y-axis represents the gene number in every pathway and process. **(B)** Gene ontology in the promoter. **(C)** Kyoto Encyclopedia of Genes and Genomes in promoter; the top 20 pathways were clustered in pathway analysis. Other results could be found in the related directory.

In our functional enrichment analysis of DMGs, we focused on CG methylation because over 90% of the identified DMGs exhibited this type of process. We categorized all the DMGs in the gene body and promoter at each time point according to GO terms and found a high degree of consistency among the various groups. Significant enrichment (*P<* 0.05) was observed for GO terms related to growth metabolism, such as “cellular process (GO: 0009987),” “metabolic process (GO: 0008152),” “cell (GO: 0005623),” and “cell part (GO: 0044464),” indicating their key role in pearl oyster growth recovery after allotransplantation. Several of the enriched GO terms were related to signal transduction, such as “membrane (GO: 0016020),” “membrane part (GO: 0044425),” and “binding (GO: 0005488)” (**Figure 5A**). These enriched GO terms were consistent in the gene body and promoter regions (**Figure 5B**), suggesting that hemolymph DMGs are crucial in regulating pearl oyster growth recovery.

To further understand the biological pathways involved in these DMGs, we performed KEGG pathway analysis and identified the top five immune-related pathways with the highest enrichment. The DMGs in the promoter region were found to be significantly involved in immune-related functions, including “Notch signaling pathway (ko 04330)” and “mRNA surveillance pathway (ko 03015).” In addition, the enriched KEGG terms included “nucleotide excision repair (ko 03420)” and “Fanconi anemia pathway (ko 03460),” which are known to effectively repair damaged DNA caused by various nonbiological stimuli. Furthermore, “Epstein–Barr virus infection (ko 05169),” which is related to preventing apoptosis of immune cells, was one of the enriched KEGG terms (**Figure 5C**) (39).

**Figure 6:**
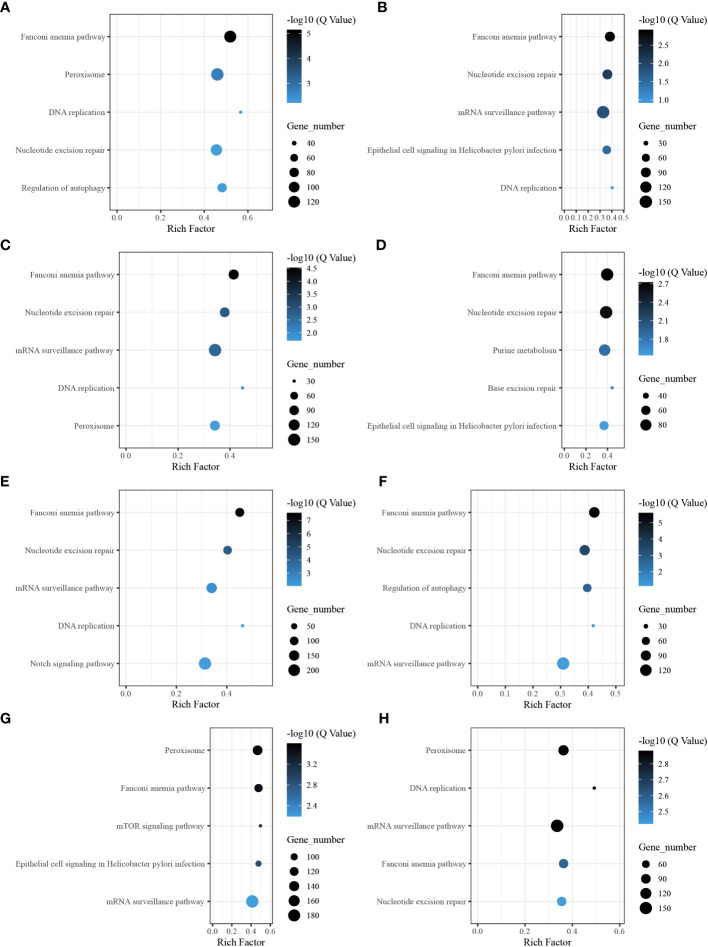
The five highest enriched immune-related pathway analyses of CG-type DMGs in gene body. **(A)** Kyoto Encyclopedia of Genes and Genomes in gene body at 6 h after grafting; the top 20 pathways were clustered in pathway analysis. Other results could be found in the related directory. **(B-H)** at 12 h, 1 d, 3 d, 6 d, 12 d, 18 d, and 30 d after grafting.

Through the pathway analysis of gene body regions, we made a noteworthy observation: the top five enriched terms varied for each time point following allotransplantation. Our analysis revealed several critical signaling pathways, including the “Fanconi anemia pathway,” “nucleotide excision repair,” “DNA replication (ko 03030),” and “Base excision repair (ko 03410),” all of which are involved in DNA excision repair. The classical immune-related pathway “mRNA surveillance pathway (ko 03015)” was enriched in all groups, except for the group of 6 h after allotransplantation (**Figures 6B–H**). Moreover, we found that the “Peroxisome (ko 04146)” pathway was significantly enriched at 6 h, 1 d, 18 d, and 30 d post-allotransplantation compared with that in the control group (**Figures 6A, C, G, H**). “Regulation of autophagy (ko 04140)” and “epithelial cell signaling in *Helicobacter pylori* infection (ko 05120),” both of which are associated with apoptosis, were also enriched (**Figures 6A, B, D, F, G**).

**Figure 7:**
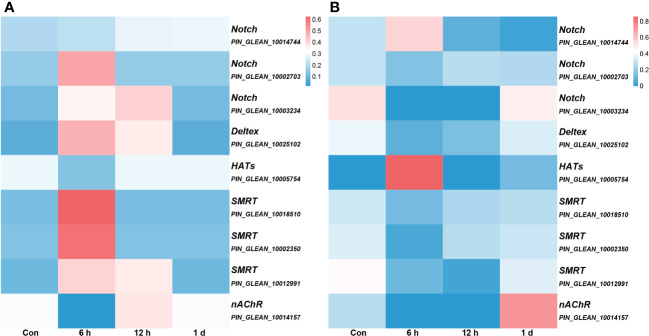
The association analysis of DNA methylation levels integrated with prior transcriptome data. All genes depicted in the figure, with the exception of *nAChR*, are associated with the Notch signaling pathway, and the gene annotation is based on KEGG annotations. **(A)** the DNA methylation levels. The color intensity of each window corresponds to the methylation level at each time point. The intensity is then normalized to the cumulative methylation level across all time points. **(B)** the transcriptome data. The color intensity of each window corresponds to the expression at each time point. Similar to **(A)**, this intensity is normalized relative to the cumulative expression across all time points.

We conducted an association analysis of DNA methylation levels of the genes in the Notch pathway with prior transcriptome data and found that when DNA methylation level in the promoter region was high, gene expression was low (**Figure 7**). This observation aligns with previous conclusions suggesting that methylation in promoter regions may influence the binding of transcription factors, consequently leading to the suppression of gene expression (12).

### 3.7 Effect of DNA methylation on promoter activity

**Figure 8:**
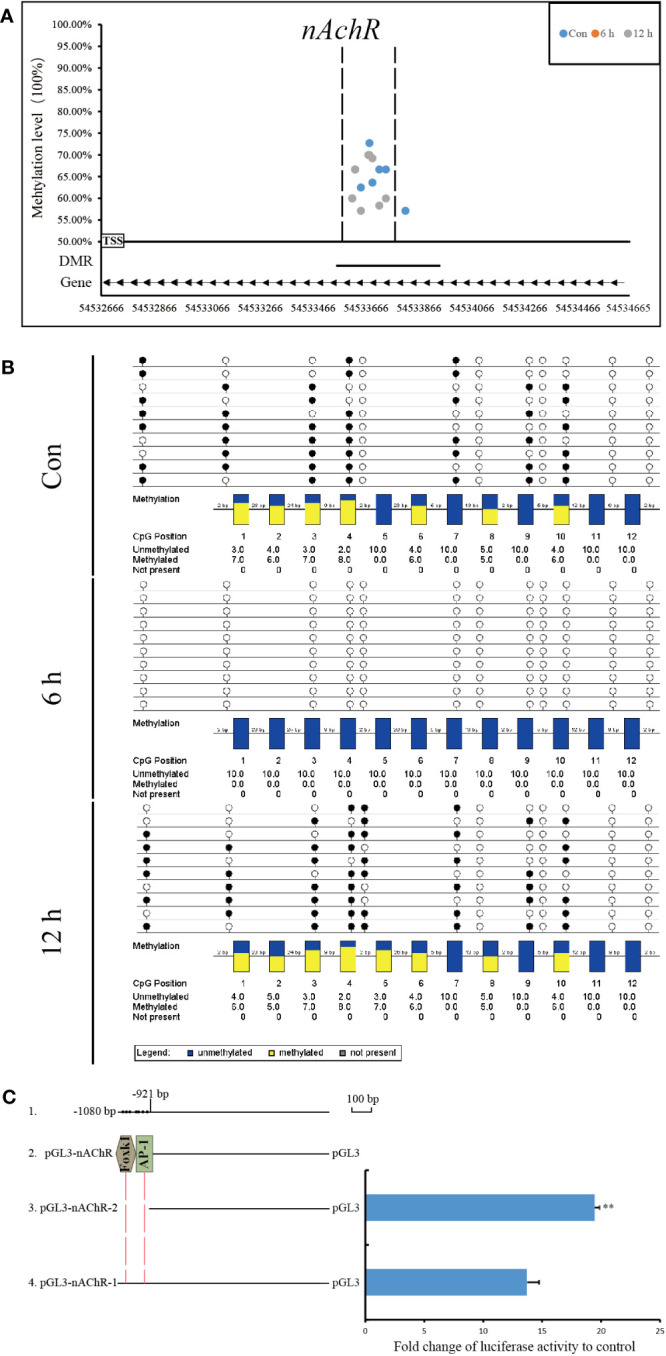
Methylation status and function analysis of the promoter region of *nAChR*. **(A)** Whole-genome bisulfite sequencing (WGBS) was used to detect methylation sites within the DMG promoter region. The horizontal axis represents the position of the promoter region of the gene in the chromosome, and the vertical axis shows the methylation level of each methylation site. The arrow direction indicates the gene’s chain orientation, where the right represents the positive chain, and the left represents the negative chain. The dashed line represents the methylation site in this region to be validated by another technique. **(B)** Bisulfite sequencing PCR was used to determine the methylation status of promoter-associated CpG residues in *nAChR* genes. The filled (black) circles correspond to methylated cytosine, the unfilled (white) circles correspond to unmethylated cytosine, and the small vertical lines without a circle correspond to the non-CpG position where there is a CpG in the genomic sequence. Each box corresponds to one CpG position in the genomic sequence. The colored bars summarize the methylation states of all sequences at that position. **(C)** Luciferase reporter constructs were used to analyze promoter expression in HEK-293T cells. Line 1 shows the location and size of promoter fragment relative to the transcription start site, as well as the location of methylation sites within the promoter region in pearl oysters. Line 2 indicates the predicted transcription factor in the dense methylation region of the promoter. Line 3, pGL3-gene promoter-2, which lacked the dense methylation region of the promoter, and Line 4, pGL3-gene promoter-1, which contained this region. The results of the luciferase reporter assay are presented in columns, with the mean and standard deviation (bars) shown. The data are representative of three independent experiments.

Compared with that at other time points, the genomic methylation level was the lowest at 6 h after allotransplantation (**Figure 3**). We found that the methylation sites in the promoter regions of *nAChR* gene exhibited significant differences at 6 h after allotransplantation compared with those in the control group, and the extent of these changes ranked prominently among the DMGs (**Supplementary Figure S4**). Methylation analysis revealed that the promoter region of the *nAChR* gene contained 6, 0, and 7 methylation sites at the three respective groups, was located between 54,533,614 and 54,533,741 in fakechr 4, and comprised 128 bp nucleotides (**Figure 8A**, **Supplementary Material 5**), and there was no methylation site in the gene body. We also observed that the *nAChR* gene expression decreased at 6 h, and increased at 1 d after allotransplantation (**Figure 7**). These results indicate that the methylation sites in the promoter regions of both genes were significantly reduced at 6 h, suggesting that the modification status of the methylation sites changes in response to stress following allotransplantation in pearl oysters.

The methylation status of CpG sites in the promoter region of the *nAChR* gene was verified by BSP. The CpG sites located between 54,533,590 and 54,533,746 in fakechr 4 were unmethylated at 6 h, and remethylation was observed at 12 h after allotransplantation (**Figure 8B**). The DNA methylation patterns of the promoter regions of *nAChR* genes by BSP were correlated with the DNA methylomic data, indicating the reliability of WGBS.

Using the transcription factor database and promoter activity prediction website, we searched for transcription factors and promoter activity regions that bind to the dense methylation region. Our analysis predicted two transcription factors that bind to the sequences of the *nAChR* promoter affected by DNA methylation in pearl oysters. To determine whether the dense methylation region affects gene transcription, we cloned 1080 bp fragments (relative to the transcription start site) spanning the dense methylation region of the *nAChR* promoter and linked them to a luciferase reporter construct (pGL3-nAChR-1) (**Figure 8C**, **Supplementary Material 6**). Dual-luciferase analysis revealed that the *nAChR* fragments had promoter activity. In particular, pGL3-nAChR-1 showed significantly decreased transcriptional activity compared with pGL3-nAChR-2. These findings suggest that the dense methylation region affects the transcriptional activity of the *nAChR* promoter in pearl oysters.

### 3.8 Differential analysis of transcription factor binding

**Figure 9:**
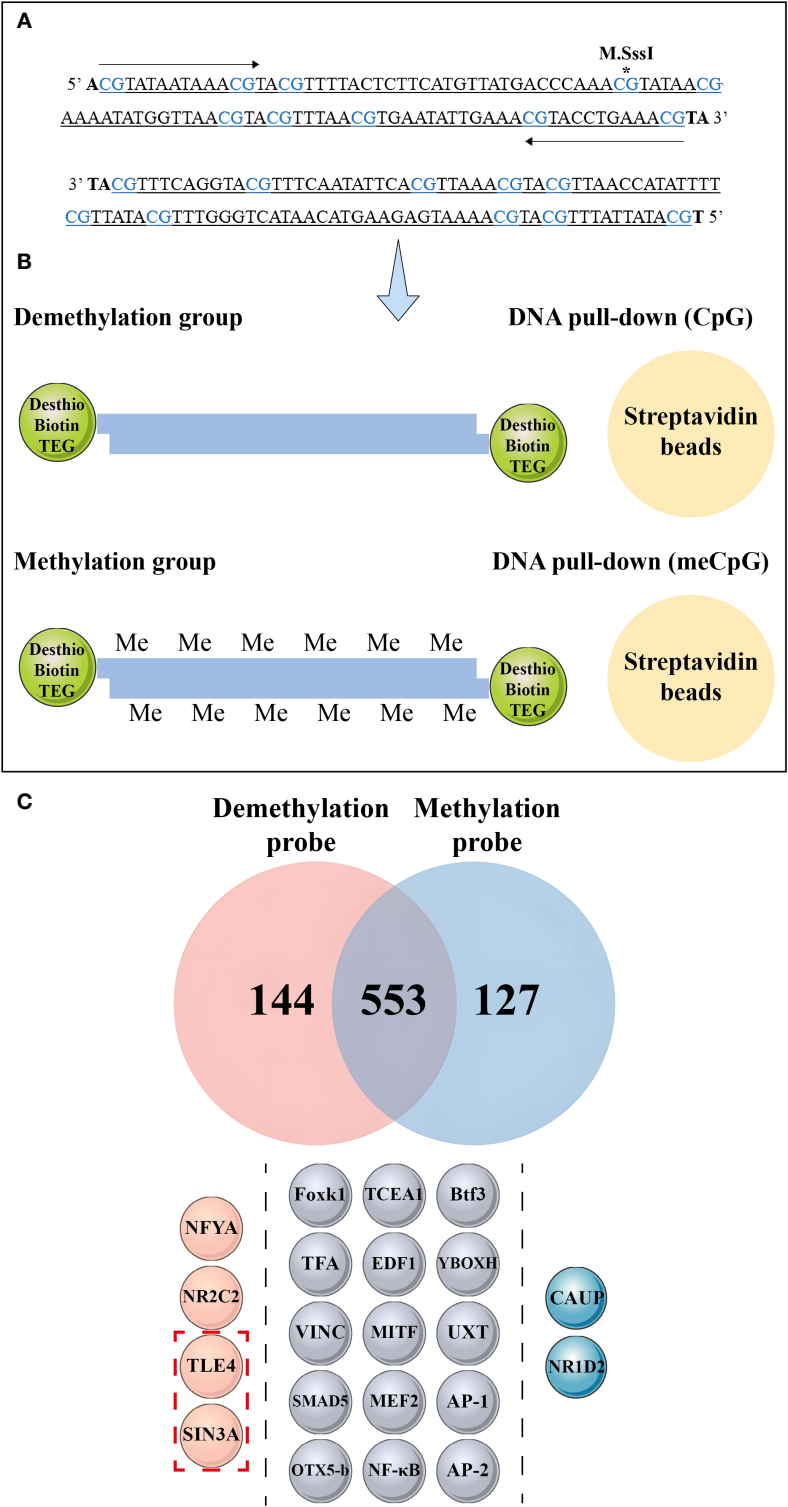
Mass spectrometry analysis and DNA pull-down-based approach to explore the effect of methylation differences on transcription factor binding. **(A)** The asterisk indicates the methylated CpG site, wherein the CpG site correspond to the 1-10th CpG sites in **Figure 8B**. **(B)** Schematic depiction of the DNA pull-down assay. **(C)** Mass spectrometry analysis reveals nuclear protein binding using different probes. The top panel shows the differential proteins bound to the methylation probe in blue and the unmethylation probe in red, while the middle panel illustrates the same protein bound to both probes. The bottom panel is divided into three sections by dashed lines, each representing different transcription factors bound by the probes. The pink spheres on the left represent transcription factors bound by non-methylation probes, the gray spheres in the middle represent transcription factors bound by both probes, and the blue spheres on the right represent transcription factors bound by methylation probes. The red dashed boxes in the bottom panel indicate the transcriptional repressors detected by mass spectrometry.

We conducted a DNA pull-down assay to investigate the effect of dense methylation regions within the *nAChR* promoter on transcription factor binding in pearl oysters. Probes were designed based on the sequence located between −921 and −1025 bp relative to the transcription start site of the *nAChR* gene. One of the probes was fully methylated using M.SssI (**Figure 9A**). In this assay, proteins from a protein extract were captured by DNA-coupled beads and analyzed by mass spectrometry. As a control experiment, fully methylated and unmethylated synthetic DNA samples were respectively used in the nuclear extracts of hemolymph in pearl oysters (**Figure 9B**).

Mass spectrometry identified 144 differential proteins bound to the demethylation probes and 127 differential proteins bound to the methylation probes, with a total of 553 proteins in common between the two probe types (**Figure 9C**, **Supplementary Material 7**). Further analysis revealed 15 transcription factors, including nuclear factor kappa-B (NF-κB), activator protein-1, Forkhead box protein K1 (Foxk1), Drosophila Mothers against decapentaplegic protein 5, and myocyte-specific enhancer factor 2A, bound to the methylation and unmethylation probes. Moreover, we detected the presence of homeobox protein caupolican and nuclear receptor subfamily 1 group D member 2-like, which only bound to the methylation probe.

We found that two transcriptional repressors, transducin-like enhancer protein 4 (TLE4) and paired amphipathic helix protein Sin3a (SIN3A), exclusively bound to the two transcription factors in the demethylation probe, namely, nuclear transcription factor Y subunit alpha and nuclear receptor subfamily 2 group C member 2.

## 4 Discussion

Researchers have discovered changes in physiological activities and molecular responses of pearl oysters after transplantation through transcriptomics (40), proteomics (41), and metabolomics (30). A variety of exogenous stimuli, including transplantation, medications, and ultraviolet radiation, can impact methylation levels, and abnormal DNA methylation is a crucial indicator of the body’s immunological function (42). In this study, we used WGBS to analyze the DNA methylation changes in pearl oysters after allotransplantation.

### 4.1 Methylation distribution pattern in pearl oyster

In pearl oyster *P. f. martensii*, approximately 92%, 2%, and 6% of the methylated cytosines were CG type, CHG type, and CHH type, respectively. Most eukaryotes, especially mouse embryonic stem cells (43) and chicken ovarian tissues (44), share this methylation distribution pattern with the greatest proportion of CG types, CHH types, and the least amount of CHG types. The highest methylation level is in fakechr 10 and the least in fakechr 15, which is consistent with our previous findings (38). We observed that the DNA methylation of CG type was demethylated at 6 h and 18 days in pearl oysters after allotransplantation, but the opposite trend was observed for the non-CG type. CG methylation and non-CG modifications are coordinately regulated by Dnmt3 and keep the same change trend in humans (45). One surprising finding is the opposite trend of DNA methylation changes between CG and non-CG types, with time or distribution position changes observed in pearl oysters. Non-CG type methylation has also been described in mammalian tissues. Its distribution across the whole genome is neither uniform nor random but is significantly enriched in several genomic features, such as gene body (46), repeat elements (47), and inactive enhancers (48). By contrast, it tends to be absent in active enhancers, promoters, and transcription factor binding sites (49). Hence, CG type and non-CG type methylations may have opposite effects on gene regulation in pearl oysters.

Repeat, also known as repetitive sequence, refers to DNA sequences that recur in the genome. Another important finding is that methylated sites are primarily found in the repeat region, with a significant variation of methylation levels at different time points. This finding is contrary to previous studies, which suggested that repeated regions are often unmethylated and have methylation levels comparable with those of nearby DNA in invertebrate species (10). This finding implied that the repeat sequence has a crucial function for gene regulation in pearl oysters after allotransplantation. Furthermore, the most prevalent sequence motifs in CHG and CHH mC sites were CAG and CAT, and both groups had the same frequencies of CHG and CHH contexts. This trend was distinct from that in mice and drosophila and comparable with that in pigs, suggesting a conservative event during evolution.

### 4.2 Critical time points for allotransplantation immunity in pearl oysters

Jiao et al. (40) discovered that two transient receptor potential cation channel genes, notch genes, and proteasome-related genes were immediately induced upon the down-regulation of cell cycle-related genes in pearl oysters 6 h after allotransplantation. The genes related to oxidation–reduction reactions, the MAPK signaling pathway, and apoptosis were induced at 6 h after allotransplantation (50). These findings are also in accordance with our observations that the number of DMRs and DMGs of Con versus 6 h was higher than that at other time points, and DNA demethylated at 6 h. We also observed the same condition 18 days after allotransplantation, reflecting the findings of Jiao et al. (40) who found that the hemocytes previously found around the transplanted mantle piece disappeared at 18 day after allotransplantation. This result implies that the immune response in the host pearl oyster will gradually disappear. Hence, these two time points are critical for pearl oyster transplant immunization, and DNA methylation is extensively involved in this process.

### 4.3 Immune-related pathways involved in DNA methylation

The GO terms “cellular process,” “cell,” and “cell part” (*P*< 0.05) were significantly enriched in gene body and promoter. Similar to the GO enrichment analysis, the KEGG pathways related to the “Epstein–Barr virus infection” were substantially enriched in promoter. Epstein–Barr virus (EBV) is also known as human herpesvirus 4 and can infect any dormant B cell *in vitro*, causing B cells to awaken and begin division (51). EBV has been connected to various human cancers and immunity where nuclear receptor co-repressor 2 and caspase 8 play crucial roles. In the human body, EBV can infect lymphocytes, mainly B cells, which are involved in apoptosis evasion, causing excessive immune cell division to cause cell cancer (52). These findings suggest that DNA methylation controls cell proliferation in the immune response brought on by allotransplantation. Several reports have shown that cellular membrane proteins are considered as main targets during virus infection (53).

The Notch signaling pathway (*P*< 0.05) was significantly enriched in the promoter at each time point, which is consistent with a previous finding that the pearl oyster can control the inflammatory response by activating the “Notch signaling pathway (40).” The Fanconi anemia pathway is a complex mechanism for the response to genotoxic insults, including three classical DNA repair pathways—homologous recombination, nucleotide excision repair, and mutagenic translesion synthesis (54). It also contains the DNA excision repair protein, which is directly associated with DNA excision repair and is necessary for the effective repair of damaged DNA. The Fanconi anemia pathway and nucleotide excision repair pathway were also enriched in the promoter. In combination with the enriched “mRNA surveillance pathway” in the promoter, we deduced that promoter DNA methylation primarily controls the downstream signaling pathway by regulating the mRNA surveillance and Notch signaling pathways; directly and indirectly manages the Fanconi anemia, nucleotide excision repair, and EBV infection pathways to control the repair of damaged DNA and the proliferation of immune cells; and regulates the overall immune response in pearl oysters after allotransplantation.

The most prominent finding from gene body analysis is the variation of significantly enriched pathways at different time points, suggesting that DNA methylation in the gene body integrally regulates immune responses through different pathways after allotransplantation. Another important finding is the enrichment of Fanconi anemia, nucleotide excision repair, DNA replication, and Base excision repair pathways in the gene body. This observation may support the hypothesis that DNA methylation primarily controls the excision repair of damaged DNA to impact the immune responses after allotransplantation.

### 4.3 Function of different methylation regions

The most common form of DNA methylation in invertebrate genomes is “gene body methylation,” and the effect of promoter methylation on gene expression completely differs from that of gene body methylation (55–57). Although promoter methylation is typically linked to transcriptional repression, gene body methylation is frequently associated with active transcription in humans and other animals (58).

This study has not elucidated the mechanism of DNA methylation regulation in the gene body due to the lack of information on higher animals. Notwithstanding the limitations, this work suggests that DNA methylation in the gene body can be involved in gene regulation in multiple ways. The regulatory functions of signaling pathways by DNA methylation of gene body in pearl oysters are an intriguing topic that could be usefully explored in further research.

The GC site’s methylation in the promoter region is frequently thought of as an epigenetic marker that prevents transcription from starting (59). By locating CG sites in CGIs, transcription factors can trigger the transcription of many genes. The transcription factor cannot bind to the promoter region and initiate transcription when the site of special recognition is methylated, thus inhibiting gene expression (60). Our study found that the promoter methylation dense regions are connected to the active promoter regions and transcription factor binding regions. Basing on the gene function and the extent of methylation differences, we chose the promoter region of the *nAChR* gene as a research object to explore the molecular regulation of DNA methylation in the promoter of pearl oysters.

### 4.4 Regulation mechanism of DNA methylation in the promoter

We examined the DNA methylation levels in three time points (Con, 6 h, and 12 h) and predicted the transcription factors binding in the methylation dense region in the *nAChR* promoter. To assess whether the methylation dense region affects *nAChR* promoter transcription, we cloned the 1080 bp fragment spanning the methylation dense region in the *nAChR* promoter and the 921 bp fragment without the methylation dense region and linked them to a luciferase reporter construct. Dual-luciferase reporter assay confirmed the possible role of the sequence of methylation dense region in transcriptional regulation of the *nAChR* promoter; significantly less transcription activity was found when the promoter fragment contained the sequence of methylation dense region. Our hypothesis is that this region of the *nAChR* promoter can attract transcription repressors binding in pearl oysters, which was confirmed by the DNA pull-down assay. We found that the methylation dense region with different methylation statuses had varying transcription factor binding abilities. The transcription repressors (p66-α, TLE4, and SIN3A) preferably bound to the methylation dense region, which was inhibited by DNA methylation.

Transcriptional repressors are a crucial component of gene regulation because they bind to the promoter region of genes and hinder the binding of RNA polymerase, thereby suppressing gene expression. TLE4, a transcriptional repressor, binds to several transcription factors and inhibits gene expression regulated by NF-κB, leading to decreased transcription activation in Wnt signaling (61). SIN3A acts as a corepressor for the RE1-silencing transcription factor, binds to the neuron-restrictive silencer element, and represses neuronal gene transcription in nonneuronal cells (62). This protein cooperates with FOXK1 to regulate cell cycle progression possibly by repressing gene expression (63). Despite the significance of transcriptional repressors in gene regulation, their role in the *nAChR* gene is poorly understood. Mass spectrometry identified NF-κB and FOXK1 in both probes from the DNA pull-down experiment, thus strongly supporting the involvement of TLE4 and SIN3A as transcriptional repressors in regulating the promoter activity of the *nAChR* gene in pearl oysters.

Some transcription factors can activate a multitude of genes by recognizing CG sites located within CpG islands. When the specific recognition sites of these transcription factors become methylated, their ability to bind to promoter regions is inhibited, thereby preventing transcriptional activation. This DNA methylation ultimately leads to a reduction in gene expression by decreasing the activity of the gene promoter region (64). In contrast to earlier findings, we hypothesized that DNA methylation increases the promoter activity of the *nAChR* gene by inhibiting the transcription repressors binding in pearl oysters. Therefore, the DNA methylation of the promoter region may also enhance the transcriptional activity and increase the immune-related gene expression, thus regulating the immune response in pearl oysters after allotransplantation. Further research is needed to verify and elaborate this phenomenon.

## 5 Conclusion

This study sheds light on the role of DNA methylation in regulating gene expression in pearl oysters subjected to allotransplantation. Our results demonstrate that the changes in methylation states within the promoter and gene body regions are important in controlling the expression of genes involved in DNA repair and cell cycle regulation. The area of the intensively methylated promoter partially coincides with the active promoter or the transcription factor binding region, explaining how the promoter methylation in pearl oysters regulates gene expression. The DNA methylation in the promoter of *nAChR* gene can affect the transcription activity by affecting the binding of different transcription factors. Overall, this study provides valuable insights into the complex regulatory mechanisms for gene expression in pearl oysters and highlights the important role of DNA methylation in this process. Future research is needed to further elucidate the precise mechanisms by which DNA methylation regulates gene expression in mollusks.

## Data availability statement

The datasets presented in this study can be found in online repositories. The names of the repository/repositories and accession number(s) can be found below: CNP0004668 (CNGB).

## Ethics statement

Ethical approval was not required for the studies on animals in accordance with the local legislation and institutional requirements because only commercially available established cell lines were used.

## Author contributions

ZG did the genome-wide DNA methylome analysis and wrote the manuscript. JY prepared the experiment samples and did the DNA pull-down. JL performed the dualluciferase assays. MY did the promoter analysis and BSP analysis. YJ, and YD designed the study and edited the manuscript. All authors contributed to the article and approved the submitted version.

## Supplementary material

Supplementary Figure 1 The proportion of three types of methylated cytosine mCG, mCHG, and mCHH. The red, blue, and green colors represent mCG, mCHG, and mCHH, respectively.

Supplementary Figure 2 Genome-wide distribution of methylation levels among different transcriptional elements. The canonical gene structure is defined by seven various features denoted by the x-axis. The length of each component was normalized and divided into equal numbers of bins. Each dot represents the mean methylation level per bin; the respective lines denote the five-bin moving average. Each feature was analyzed separately for the numbers listed in the table below the figure. The green vertical line indicates the mean location of the transcription start sites.

Supplementary Figure 3 Sequence preferences for methylation in CG, CHG, and CHH contexts. The horizontal axis represents the base position, methylated cytosine is in the fourth position, while the vertical axis indicates the entropy of the base. The base sequence from top to bottom suggests the preference degree from high to low.

Supplementary Figure 4 Distribution of methylation difference. The boxplot displays the variation in methylation levels between the Con and 6 h groups, within the gene body and promoter regions. The vertical axis represents the disparity in average DNA methylation levels between these two groups.
