# Supplementary figures and images for: Whole-genome bisulfite sequencing reveals the function of DNA methylation in the allotransplantation immunity of pearl oysters
Source: Front Immunol. 2023 Oct 3;14:1247544. doi: 10.3389/fimmu.2023.1247544 (PMC10579932; doi:10.3389/fimmu.2023.1247544)

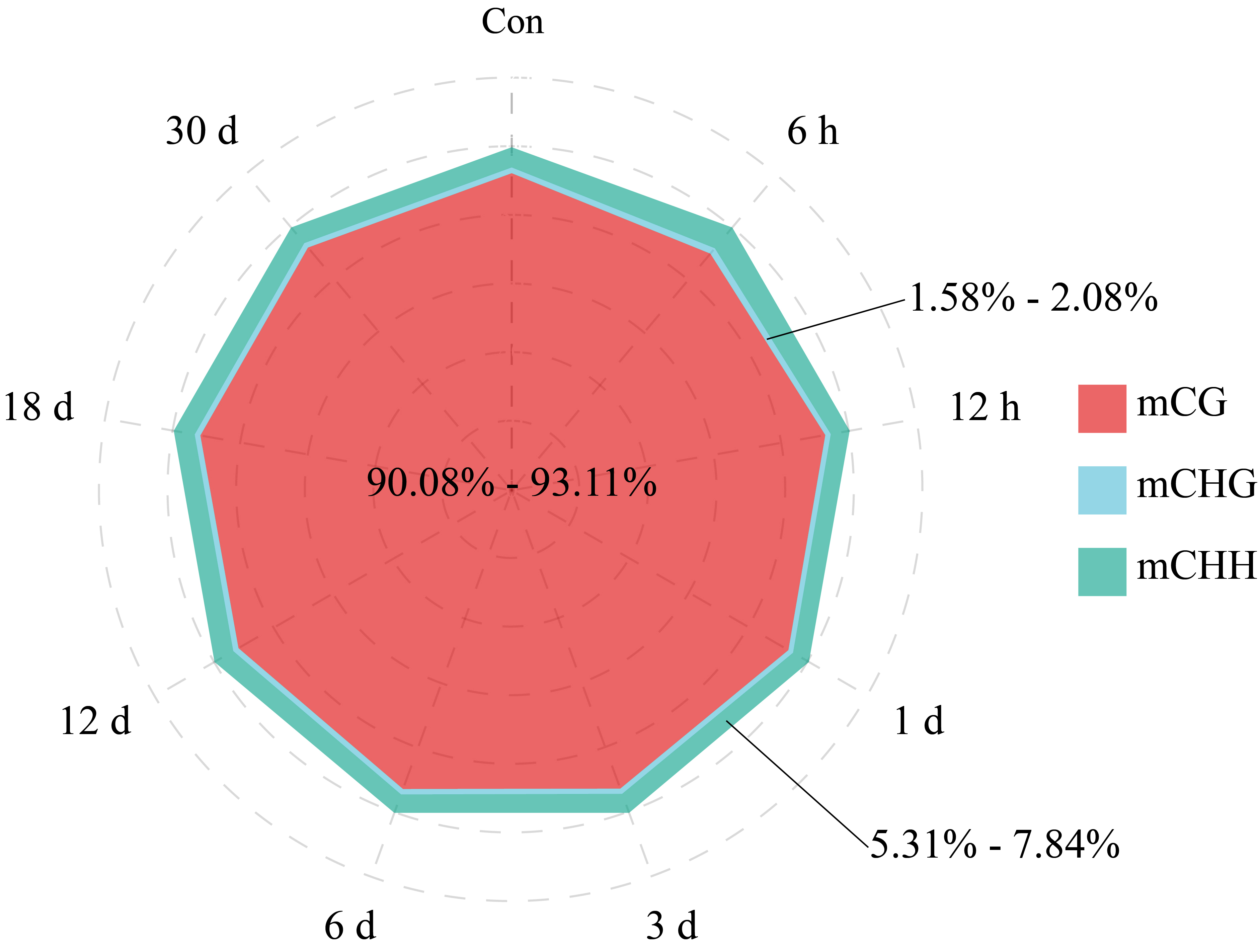

Supplement: Supplementary Figure 1 — The proportion of three types of methylated cytosine mCG, mCHG, and mCHH. The red, blue, and green colors represent mCG, mCHG, and mCHH, respectively. [file Image_1.jpeg]

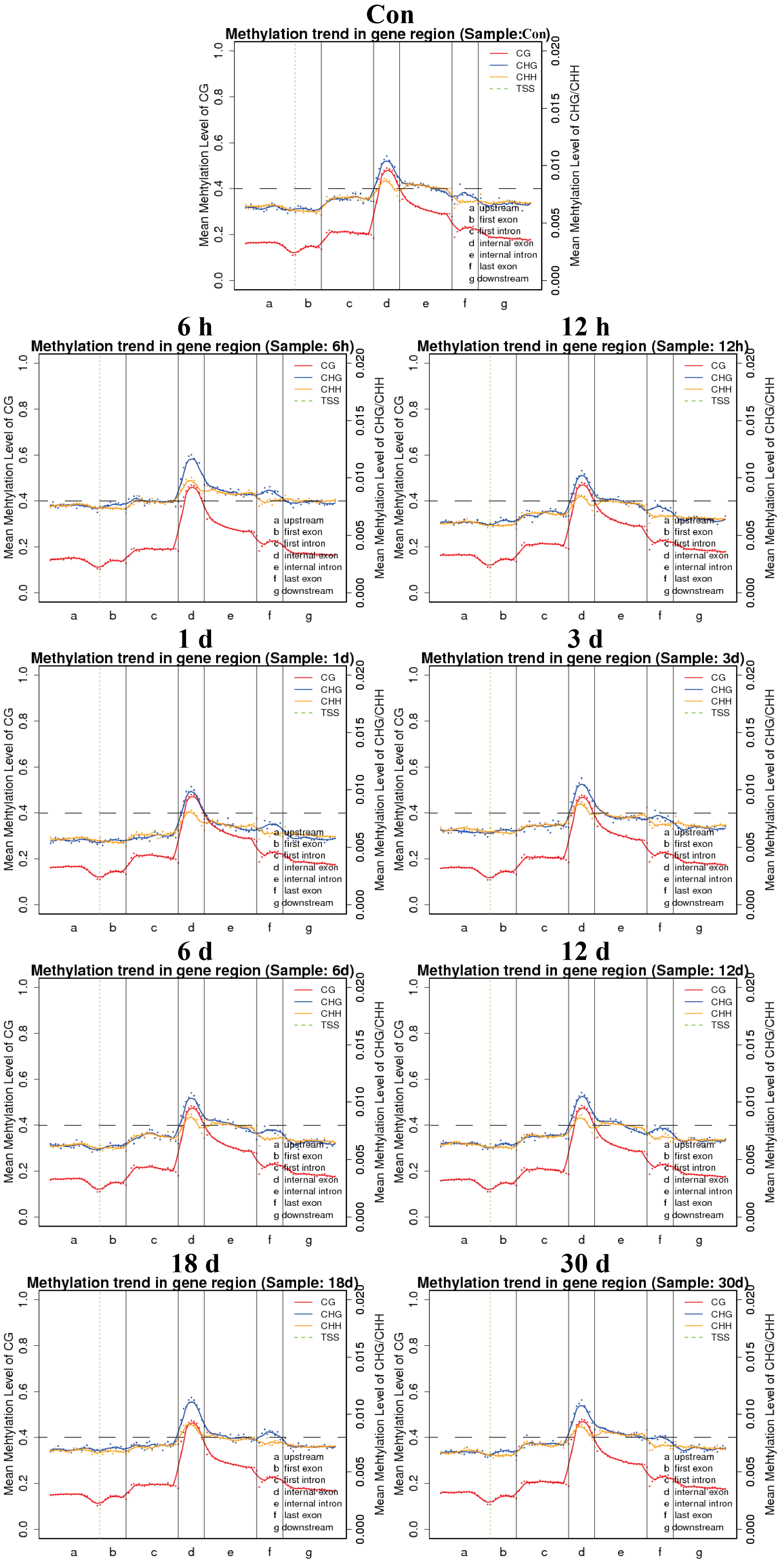

Supplement: Supplementary Figure 2 — Genome-wide distribution of methylation levels among different transcriptional elements. The canonical gene structure is defined by seven various features denoted by the x-axis. The length of each component was normalized and divided into equal numbers of bins. Each dot represents the mean methylation level per bin; the respective lines denote the five-bin moving average. Each feature was analyzed separately for the numbers listed in the table below the figure. The green vertical line indicates the mean location of the transcription start sites. [file Image_2.jpeg]

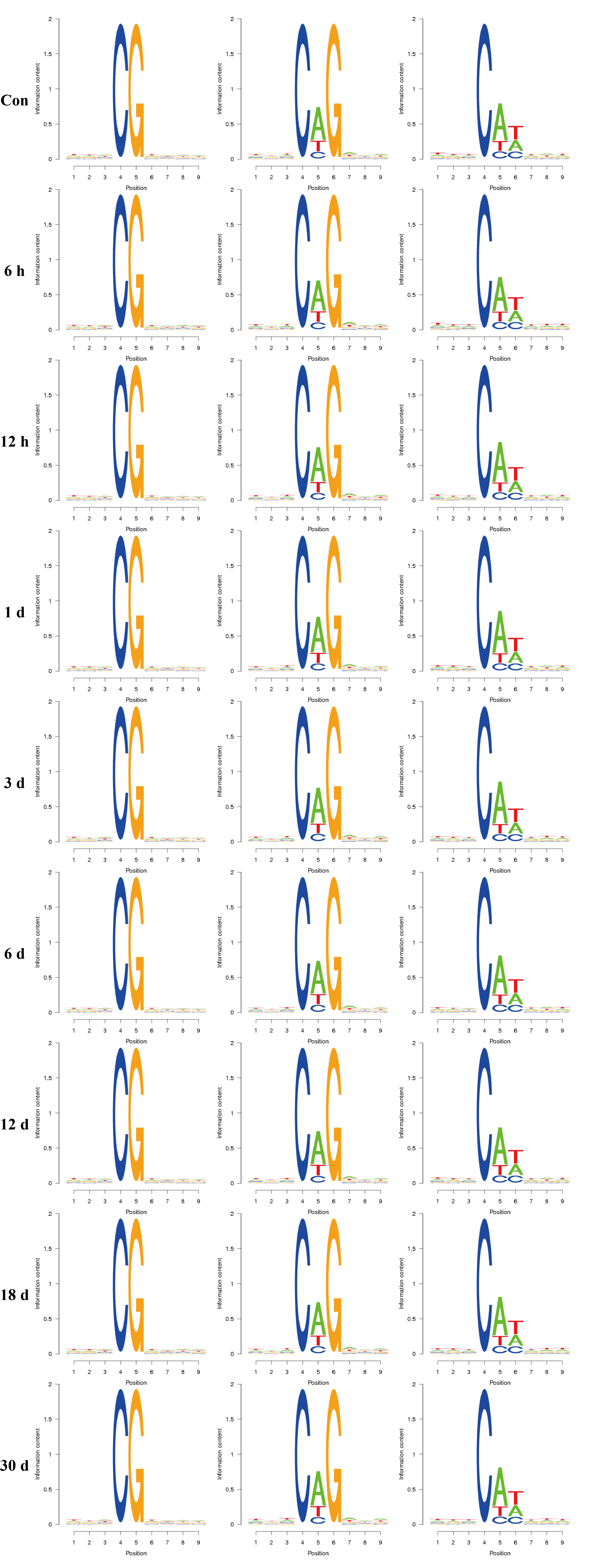

Supplement: Supplementary Figure 3 — Sequence preferences for methylation in CG, CHG, and CHH contexts. The horizontal axis represents the base position, methylated cytosine is in the fourth position, while the vertical axis indicates the entropy of the base. The base sequence from top to bottom suggests the preference degree from high to low. [file Image_3.jpeg]

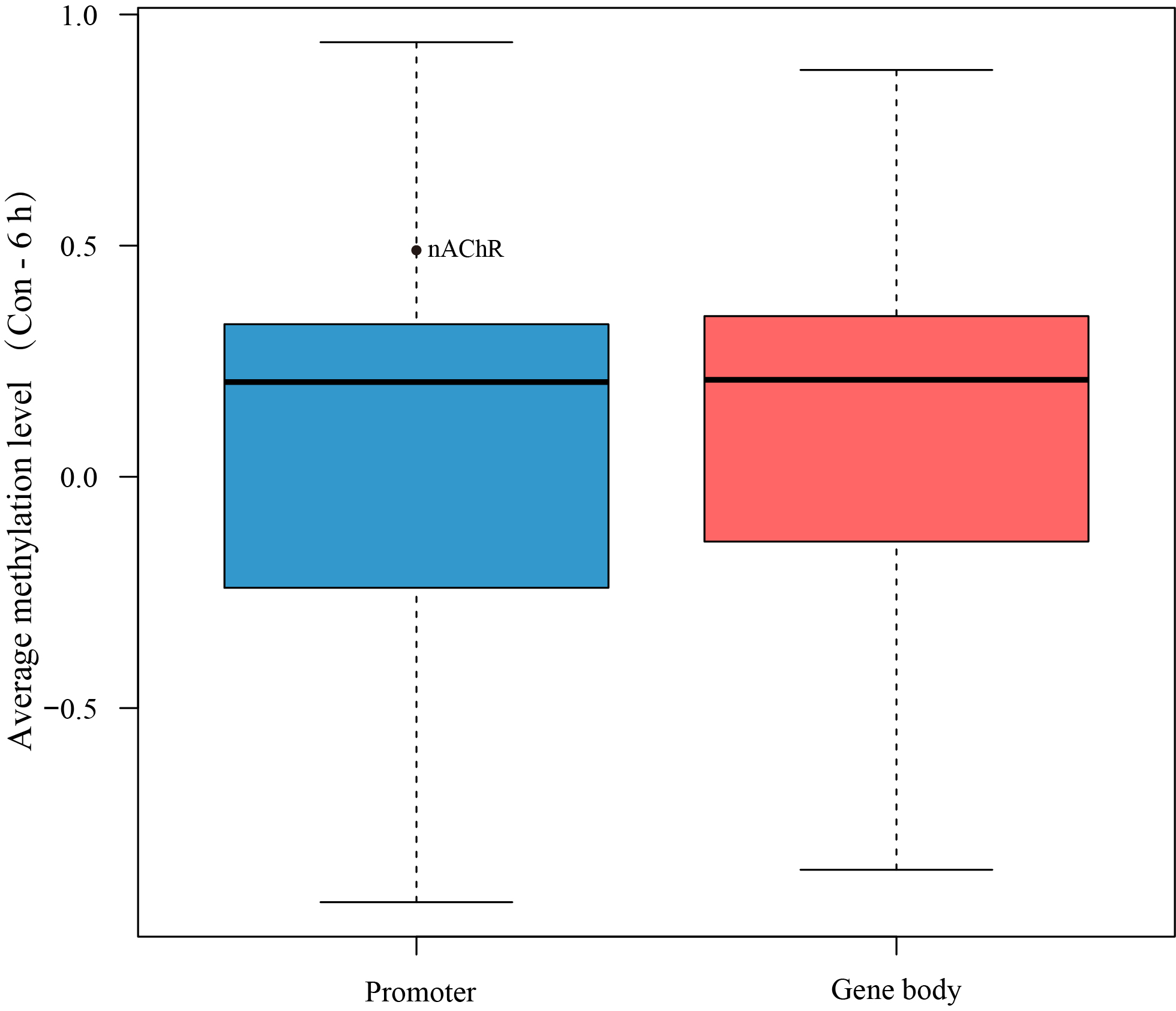

Supplement: Supplementary Figure 4 — Distribution of methylation difference. The boxplot displays the variation in methylation levels between the Con and 6 h groups, within the gene body and promoter regions. The vertical axis represents the disparity in average DNA methylation levels between these two groups. [file Image_4.jpeg]
